# Supplementary material for: Terpinen-4-ol, the Main Bioactive Component of Tea Tree Oil, as an Innovative Antimicrobial Agent against Legionella pneumophila
Source: Pathogens. 2022 Jun 14;11(6):682. doi: 10.3390/pathogens11060682 (PMC9229490; doi:10.3390/pathogens11060682)
Supplement: Supplementary file 1 [file pathogens-11-00682-s001.zip › pathogens-1744154-supplementary.pdf]

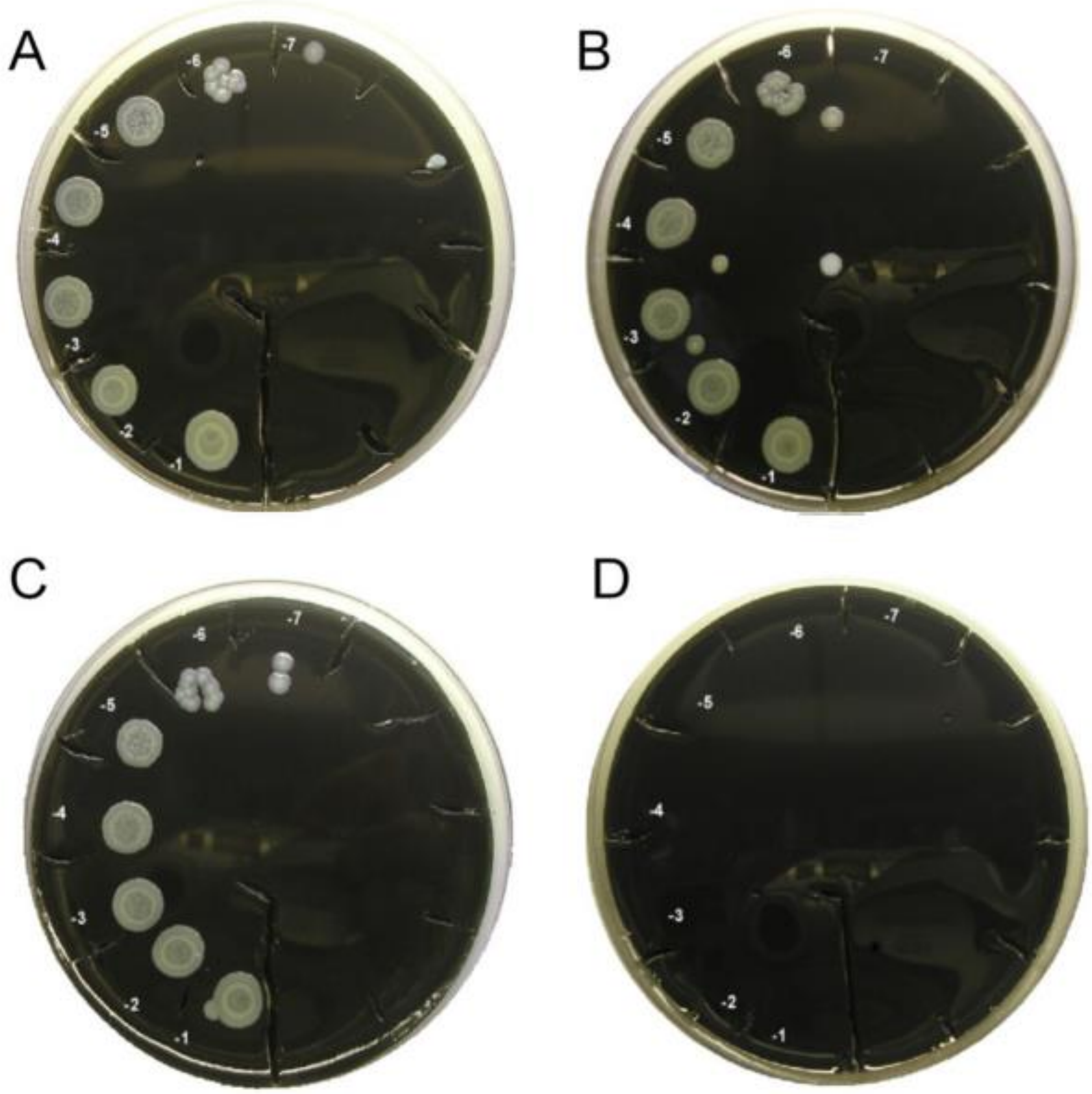

Figure S1. Legionella pneumophila sg1 ATCC 33152 growth in T-4-ol treated and untreated samples at different temperatures for SEM experiments. A: Untreated controls growth at T0; B, C: Untreated controls growth after one hour at 40° and 45° C; D: Lp treated with T-4-ol (0.42% v/v) or TTO (1%v/v) after one hour at 40° and 45° C (only a plate is represented
